# Investigated the safety of intra-renal arterial transfusion of autologous CD34+ cells and time courses of creatinine levels, endothelial dysfunction biomarkers and micro-RNAs in chronic kidney disease patients-phase I clinical trial

**DOI:** 10.18632/oncotarget.14831

**Published:** 2017-01-27

**Authors:** Mel S. Lee, Fan-Yen Lee, Yung-Lung Chen, Pei-Hsun Sung, Hsin-Ju Chiang, Kuan-Hung Chen, Tien-Hung Huang, Yi-Ling Chen, John Y. Chiang, Tsung-Cheng Yin, Hsueh-Wen Chang, Hon-Kan Yip

**Affiliations:** ^1^ Department of Orthopedics, Kaohsiung Chang Gung Memorial Hospital and Chang Gung University College of Medicine, Kaohsiung, Taiwan; ^2^ Division of Thoracic and Cardiovascular Surgery, Department of Surgery, Kaohsiung Chang Gung Memorial Hospital and Chang Gung University College of Medicine, Kaohsiung, Taiwan; ^3^ Division of Cardiology, Department of Internal Medicine, Kaohsiung Chang Gung Memorial Hospital and Chang Gung University College of Medicine, Kaohsiung, Taiwan; ^4^ Department of Obstetrics and Gynecology, Kaohsiung Chang Gung Memorial Hospital and Chang Gung University College of Medicine, Kaohsiung, Taiwan; ^5^ Department of Anesthesiology, Kaohsiung Chang Gung Memorial Hospital and Chang Gung University College of Medicine, Kaohsiung, Taiwan; ^6^ Department of Computer Science and Engineering, National Sun Yat-Sen University, Kaohsiung, Taiwan; ^7^ Department of Healthcare Administration and Medical Informatics, Kaohsiung Medical University, Kaohsiung, Taiwan; ^8^ Department of Biological Sciences, National Sun Yat-Sen University, Kaohsiung, Taiwan; ^9^ Institute for Translational Research in Biomedicine, Kaohsiung Chang Gung Memorial Hospital and Chang Gung University College of Medicine, Kaohsiung, Taiwan; ^10^ Center for Shockwave Medicine and Tissue Engineering, Kaohsiung Chang Gung Memorial Hospital and Chang Gung University College of Medicine, Kaohsiung, Taiwan; ^11^ Department of Nursing, Asia University, Taichung, Taiwan; ^12^ Department of Medical Research, China Medical University Hospital, China Medical University, Taichung, Taiwan

**Keywords:** chronic kidney disease, endothelial dysfunction, endothelial progenitor cells, autologous transfusion of CD34+ cells into renal artery

## Abstract

This was a phase I clinical trial to investigate the safety of autologous peripheral-blood-derived CD34+ cell therapy for patients with chronic kidney disease (CKD-treatment) (i.e., at Stages III and IV). Between November 2014 and October 2015, a total of 10 study patients were prospectively enrolled into this phase I trial. Patients who failed to enroll into the trial in the initial state of eligibility assessment were served as CKD-control group (*n* = 9). The health-control group was composed of 10 volunteers for the purposes of comparing (1) circulation level of endothelial progenitor cells (EPCs), (2) angiogenesis ability, and (3) anti-apoptotic miRNAs between healthy subjects and CKD patients. CD34+ cells (5.0 × 10^7^) were transfused into right-renal artery after subcutaneous G-CSF injection (5μg/kg/twice a day for 4 days). Circulating EPC number, angiogenesis capacity (i.e., Matrigel assay) and anti-apoptotic miRNAs (miR-374a-5p/miR-19a-3p/ miR-106b-5p/miR-26b-5p/ miR-20a-5p) were significantly lower in CKD patients than in healthy subjects (all *p* < 0.001). Flow-cytometric analysis of renal-vein blood samplings (i.e., at 0/5/10/30 mins after cell transfusion) showed the EPC level was significantly progressively increased (*p* < 0.001). Procedural safety was 100% with all patients uneventfully discharged and one-year survival rate was 100%. The paired-*t* test showed serum creatinine maintained the same level between the baseline and at the end of one-year follow-up (all *p* > 0.4), whereas the net increase between initial and final creatinine level was higher in CKD-control than in CKD-treatment. In conclusion, CD34+ cell therapy was safe and maintained the renal function in stationary state at the end of study period.

## INTRODUCTION

Chronic kidney disease (CKD) with vast known and unknown causal etiologies clearly established [1–6] remains the major and growing contributor to health care burden worldwide [7–9]. Abundant data from clinical observational studies have revealed that CKD contributed high morbidity and mortality in hospitalized patients, especially in those CKD patients with co-existing cardiovascular disease (i.e., cardiorenal syndrome) [10–14]. Surprisingly, despite the state-of-the-art therapeutic and advanced pharmaceutical strategies, such as the uses of angiotensin converting enzyme inhibitor (ACEI), angiotensin II type I receptor blockade (ARB), and direct renin inhibitor (DRI), as well as good education, and renewed guideline for CKD precise management, progressive deterioration of renal function is frequently observed, subsequently leading to the adverse development of end-stage renal disease (ESRD) in CKD patients [15–20].

Through more than several-decade keen investigation, the aetiology of CKD has been clearly shown to be divergent and the mechanisms involved are complicated. Endothelial cell dysfunction in the arterioles, followed by propagation and the development of obstructive atherosclerosis [21–25], has been implicated as one of the major aetiology in the pathogenesis of the CKD disease. In particular, inflammatory reactions, fibrosis formation and generations of oxidative stress and reactive oxygen species (ROS) have been reported as the principal mechanisms involved in the disease [26–31]. Taken into account the variety of aetiologies and the intricate mechanisms involved, satisfactory treatment of the disease with a single therapeutic strategy would be impossible. Therefore, finding an innovative therapeutic strategy with relatively broad-spectrum effect for preservation and improvement of renal microvasculature/endothelial function and renal function is a topic of utmost importance.

Results from clinical observational studies [32, 33], including our recent clinical study [34], have shown that the number and function of endothelial progenitor cells (EPCs) decreased significantly in patients with CKD. On the other hand, numerous experimental studies have shown that stem cell therapy improves ischemia-induced organ dysfunction through angiogenesis and restoring blood flow in the ischemic area [35–37]. Additionally, growing data from clinical trials [38], including our recent study [39], have shown that both circulatory derived and bone marrow derived CD34+ stem cell therapy remarkably improved angina and ischemia-related left ventricular dysfunction, and restored the microvascular blood flow.

Recently, our experimental study demonstrated that peripheral blood-derived EPC therapy could impede the deterioration of CKD induced by 5/6 nephrectomy in rats [37]. The results of our animal model study [37] and clinical trial [39] encouraged the use of circulatory-derived autologous stem cell therapy for patients with CKD. Accordingly, a phase I clinical trial employing autologous CD34+ cell to treated patients with CKD stages III and IV was performed with two essential purposes as: (1) to investigate the safety of autologous administration of peripheral blood-derived CD34+ cells to CKD patients, and (2) to analyze endothelial dysfunction elements, focusing on the angiogenesis (i.e., by Matrigel assay) and circulating anti-apoptotic micro-RNAs.

## RESULTS

### Comparison of serial changes of creatinine levels between CKD-control group and CKD-treatment group during one-year follow-up, and the results of renal ultrasound and clinical follow-up (Figures 1 and 2 and Table 1)

**Figure 1:**
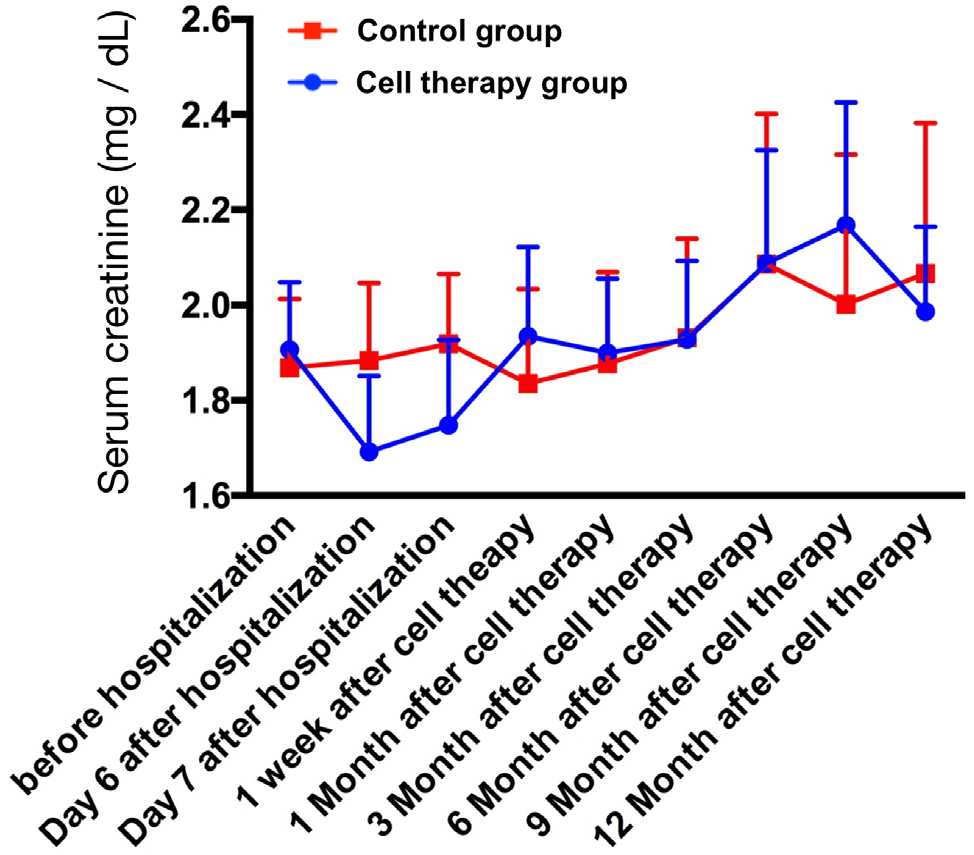
The time courses of serum level of creatinine among the chronic kidney disease (CKD)-treatment (*n* = 10) (i.e CD34+ cell therapy) and CKD-control (*n* = 9) patients. The relative lower creatinine level at day 6 and day 7 of hospitalization could be due to the normal saline hydration therapy.

**Figure 2:**
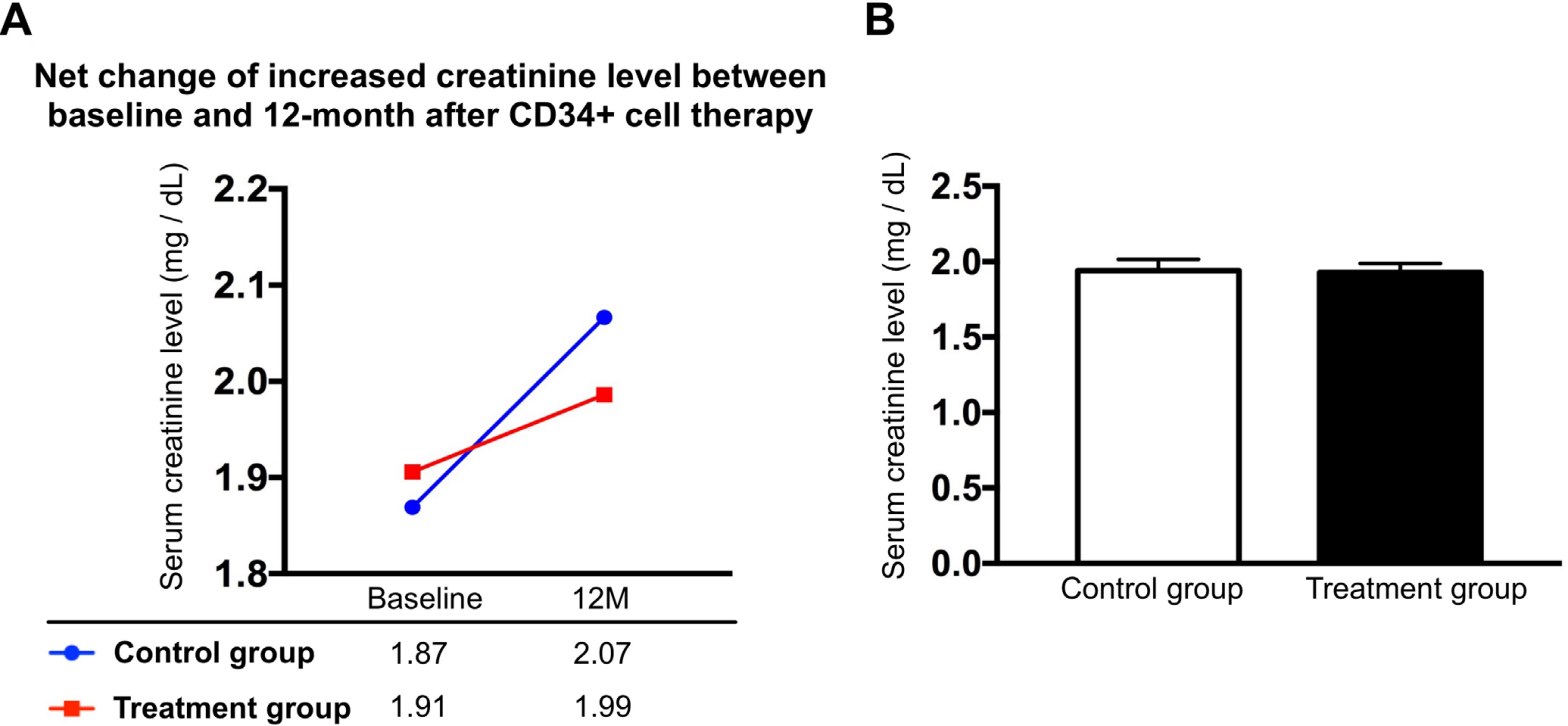
Comparison of an increased net change (Δ) of creatinine level between CKD-treatment and CKD-control groups with respect to the time intervals between baseline and 12th month, and mean summation of serial changes of creatinine level in CKD-treatment (*n* = 10) and CKD-control groups (*n* = 9) (**A**) An increase in net change of creatinine level (i.e., between baseline and 12th month) was noted little bit higher in CKD-control group than in CKD-treatment group. (**B**) Mean summation of serial changes of creatinine level, CKD-treatment vs. CKD-control, *p* = 0.866.

Ten blood samplings were consecutively drawn in both CKD-control group and CKD-treatment group (i.e., autologous CD34+ cells therapy) during one-year follow-up after CD34+ cell therapy. The serial changes of circulating levels in both CKD-control group and CKD-treatment group were plotted in Figure 1. The results showed that the baseline level of serum creatinine (i.e., prior to CD34+ cell therapy) remained the same level between CKD-treatment and CKD-control groups (1.91 ± 0.45 vs. 1.87 ± 0.48, *p* = 0.23) (Figure 2A). Additionally, by the end of study period (i.e., at the end of on-year follow-up), the serum creatinine was also similar between both groups (1.98 ± 0.69 vs. 2.01 ± 1.05, *p* = 0.68) (Figure 2A). Furthermore, the mean summation of all creatinine levels (i.e., from the baseline to the final time interval of creatinine levels submitted) also maintained the same between the CKD-control and the CKD-treatment groups (1.93 ± 0.59 vs. 1.94 ± 0.59, *p* = 0.881) (Figure 2B). However, the net change of creatinine level between baseline and the end of study period was relative lower in CKD-treatment group than in CKD-control counterpart [1.98–1.91/1.98 (3.5%) vs. 2.07–1.87/2.07 (9.7%), *p* = 0.667], implicating that CD34+ cell therapy may ameliorate the deterioration of renal function in CKD patients (Figure 2A).

The serial follow-ups (i.e., at baseline, and 1, 3, 6 and 12 months after CD34+ cell treatment) of renal ultrasound showed no identifiable abnormality of anatomical/structural change of kidney or tumorigenesis. Additionally, the kidney size (i.e., long axis and short axis) did not differ between the baseline and the end of study period (i.e., the 12-month follow up) (Table 1).

**Table 1:** Serial ultrasound results of right kidney after CD34+ cell therapy (*n* = 10)

Variables	baseline	1-M	3-M	6-M	12-M	*p*-value
Length (cm)*	10.9 ± 0.8	11.0 ± 0.8	10.9 ± 0.7	10.9 ± 0.8	10.7 ± 0.8	0.882
Width (cm)*	5.4 ± 0.6	5.4 ± 0.6	5.5 ± 0.6	5.3 ± 0.6	5.3 ± 0.5	0.867
Grade of cortical echogenicity	1.2 ± 0.35	1.2 ± 0.35	1.2 ± 0.35	1.1 ± 0.21	1.2 ± 0.34	0.698
Parenchymal thickness (cm)	1.4 ± 0.15	1.4 ± 0.10	1.4 ± 0.16	1.3 ± 0.18	1.4 ± 0.11	0.867
dilated pelvicalyceal systems†	0	0	0	0	0	–
Mass in right kidney†	0	0	0	0	0	–

Data expression as mean ± SD.

M = month.

* indicates the long and short axis of kidney size, respectively.

† indicates none.

One-year survival rate was 100% in both study and control patients. However, two study patients who experienced acute non-ST segment elevation myocardial infarction (non-STEMI) (Killip-1 in one patient and Killip-3 in the other patient, respectively) underwent primary coronary intervention. The STEMI patient with Killip-3 upon presentation at the time interval after 12 months of CD34+ cell therapy developed end-stage renal disease on regular hemodialysis after primary coronary intervention mainly due to contrast media-induced nephrotoxicity. These two patients remain with regular follow-up at outpatient department.

### The serial changes of BUN level and ratios of urine total protein and urine albumin to urine creatinine in CKD-treatment group during one-year follow-up (Table 2)

The serum level of BUN was significantly lower at one week after CD34+ cell therapy than in the other times intervals, but it showed not difference among the other time intervals. Additionally, the ratios of urine albumin and total protein to urine creatinine were lower at the end of study period (i.e., at 12-month follow-up) than in the other time intervals. However, they exhibited no difference among the other time intervals. On the other hand, the creatinine clearance (Ccr) rate did not differ among all the time intervals, implicating the CD34+ therapy might maintain the Ccr rate at a stationary status at the end of study period.

**Table 2:** Time courses of BUN, ratios of urine albumin and urine protein to urine creatinine and creatinine clearance rate (*n* = 10)

Variables	Baseline	1-week	1-month	3-month	6-month	9-month	12-month	*p*-value
BUN (mg/dL)	29.7 ± 11.7^a^	20.9 ± 12.4^b^	31.8 ± 5.1^a^	33.8 ± 13.2^a^	35.8 ± 21.3^a^	25.6 ± 12.2^a,b^	35.1 ± 26.3^a^	< 0.01
Ualb-cre ratio	885 ± 1271^a,b^	951 ± 1370^a^	1486 ± 2145^a^	1359 ± 2024^a^	1303 ± 2356^a^	1385 ± 3361^a^	221 ± 416^b^	< 0.005
TP(u)-cre ratio	2484 ± 3788^a^	1221 ± 2050^a^	2724 ± 3677^a^	2238 ± 3422^a^	2265 ± 4055^a^	2482 ± 5095^a^	366 ± 778^b^	< 0.01
Ccr (ml/min)	45.3 ± 14.6	52.5 ± 19.3	47.5 ± 20.8	46.6 ± 17.2	44.1 ± 18.6	43.7 ± 20.3	45.8 ± 23.8	> 0.6

Data are expressed as mean ± SD.

Abbreviations: BUN = blood urine nitrogen; Ualb-cre ratio = urine albumin to urine creatinine ratio; TP(u)-cre ratio = urine total protein to urine creatinine ratio; Ccr = creatinine clearance rate.

Letters (a,b) indicate significant difference (at 0.05 level) by Wilcoxon's signed-rank test with multiple comparison procedure.

### Baseline characteristics and comparison of circulating number of EPCs between healthy subjects and CKD-treated patients, and before vs. after G-CSF treatment among CKD-treated patients (Figures 3 and 4)

The age distribution was significantly younger in health-control group as compared to CKD-treated group (29.0 ± 3.3 vs. 64.6 ± 17.8, *p* < 0.01). On the other hand, no difference existed in gender distribution between these two groups [for male gender: health-control 70% (7/10) vs. CKD-treatment 80% (8/10), *p* = 0.606].

**Figure 3:**
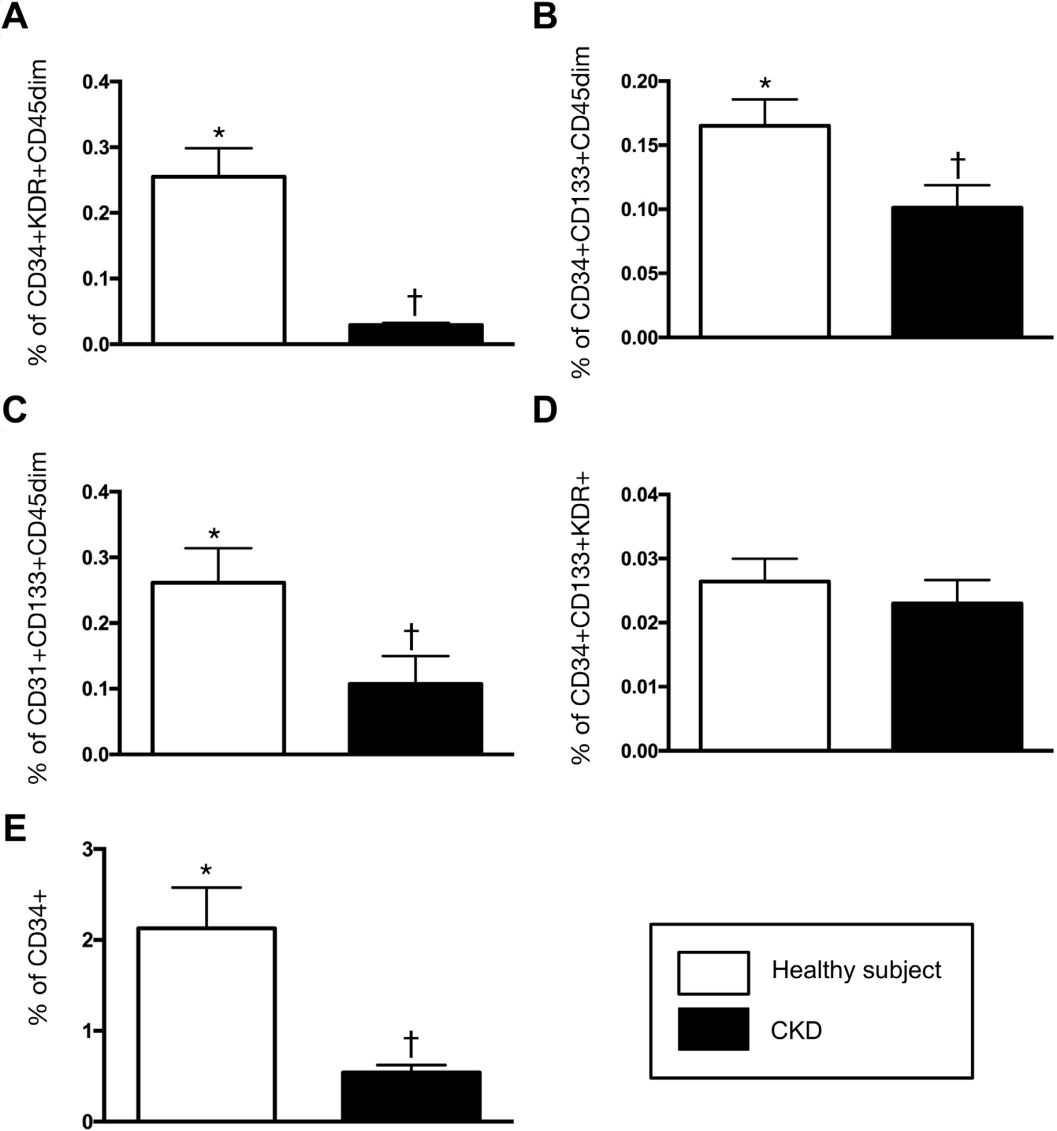
Comparison of circulating level of endothelial progenitor cells (EPCs) and hematopoietic stem cell (CD34+) between CDK (*n* = 10) and health-control (*n* = 10) groups (**A**) CD34+KDR+CD45^dim^ (%), * vs. †, *p* < 0.0001. (**B**) CD34+CD133+CD45 ^dim^ (%), * vs. †, *p* < 0.002. (**C**) CD31+CD133+CD45 ^dim^ (%), * vs. †, p0.017. (**D**) CD34+CD133+KDR+ (%), CKD vs. health subjects, *p* = 0.255. (**E**) CD34+ (%), * vs. †, p0.002. All statistical analyses were performed by Wilcoxon's rank sum test.

As expected, the baseline (i.e., prior to G-CSF treatment) circulating levels of EPCs (i.e., CD34+KDR+CD45^dim^, CD34+CD133+CD45 ^dim^ and CD31+CD133+CD45 ^dim^) and hematopoietic stem cell (HSC) (CD34+) were significantly lower in CDK-treated group than in the healthy counterpart (Figure 3).

**Figure 4:**
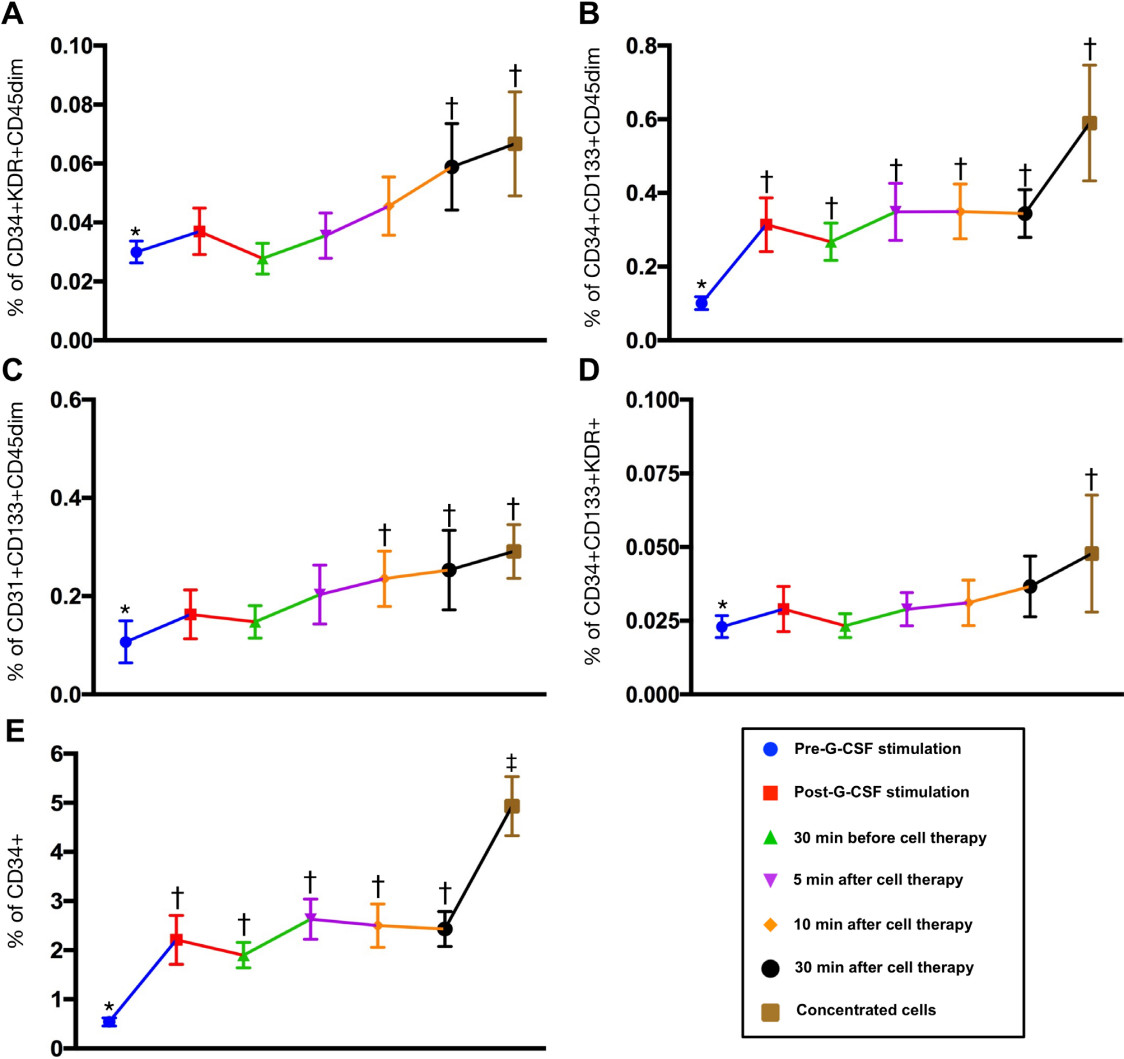
Time courses of endothelial progenitor cells (EPCs) and hematopoietic stem cell (CD34+) (**A)** CD34+KDR+CD45^dim^ (%), * vs. †, *p* < 0.0001. (**B)** CD34+CD133+CD45 ^dim^ (%), * vs. †, *p* < 0.002. **(C)** CD31+CD133+CD45 ^dim^ (%), * vs. †, p0.017. (**D)** CD34+CD133+KDR+ (%), CKD vs. health subjects, *p* = 0.255. (**E)** CD34+ (%), * vs. other groups with different symbols (†, ‡), *p* < 0.0001. Statistical analysis using Friedman test followed by Wilcoxon's signed-rank test with multiple comparison procedure. Symbols (*, †, ‡) indicate significance (at 0.05 level).

To elucidate the shedding rate of EPCs from renal vein after CD34+ cell transfusion, time courses of EPC and HSC measurement were performed by flow cytometry. The results showed that the EPCs and HSC were continuously drained from renal vein to circulation after intra-renal artery transfusion (Figure 4). Additionally, among the CKD-treated patients, the circulating level of EPCs and HSC were notably higher after G-CSF treatment than that prior to G-CSF treatment (Figure 4). This finding suggests that G-CSF treatment allowed the EPC and HSC homing from bone marrow to circulation.

### Determinant of angiogenesis by Matrigel assay in CKD-treatment and health-control groups (Figure 5)

The results of Matrigel assay demonstrated that the angiogenesis (i.e., including cluster formation, tubular length, and network formation) capacity prior to G-CSF treatment was significantly lower in CKD-treated group than in health-control group. However, the angiogenesis capacity was significantly enhanced in CKD-treated group after receiving the G-CSF treatment.

### Comparison of circulating microRNA expressions between health-control subjects and in CKD-treated patients (Figures 6 and 7)

Unsupervised hierarchical clustering was done on the real-time qPCR array for investigating the relationships among miRNAs and CKD that was shown in the format of clustergram (Figure 6A). Total 84 miRNAs were illustrated, among these, 18 miRNAs that were the most significantly downregulated in CKD patients (> 2 folds) and no upregulated miRNA was founded (Figure 6B). For the convincing evidence, top five downregulated miRNAs, including miR-374a-5p (with biological processes of proliferation, anti-apoptosis and migration), miR-19a-3p (with biological processes of angiogenesis, glycogenesis and anti-apoptosis), miR-106b-5p (with biological processes of proliferation, migration and anti-apoptosis), miR-26b-5p (with biological processes of anti-autophagy, migration and anti-apoptosis) and miR-20a-5p (with biological processes of migration, radioresistance and anti-apoptosis), were the candidates for investigation. A diverging change of circulating levels of these five miRNAs was identified before and after G-CSF treatments (Figure 7A–7E). Additionally, the levels of some miRNAs were found to be significantly increased in some CKD-treated patients after receiving G-CSF treatment. However, the expressions of these five miRNAs were significantly lower in CKD-treated patients than in health-control subjects, implicating that all the CKD-treated patients had notably lower intrinsic capacities of migratory, proliferation and anti-apoptosis (Figure 7F–7J).

**Figure 5:**
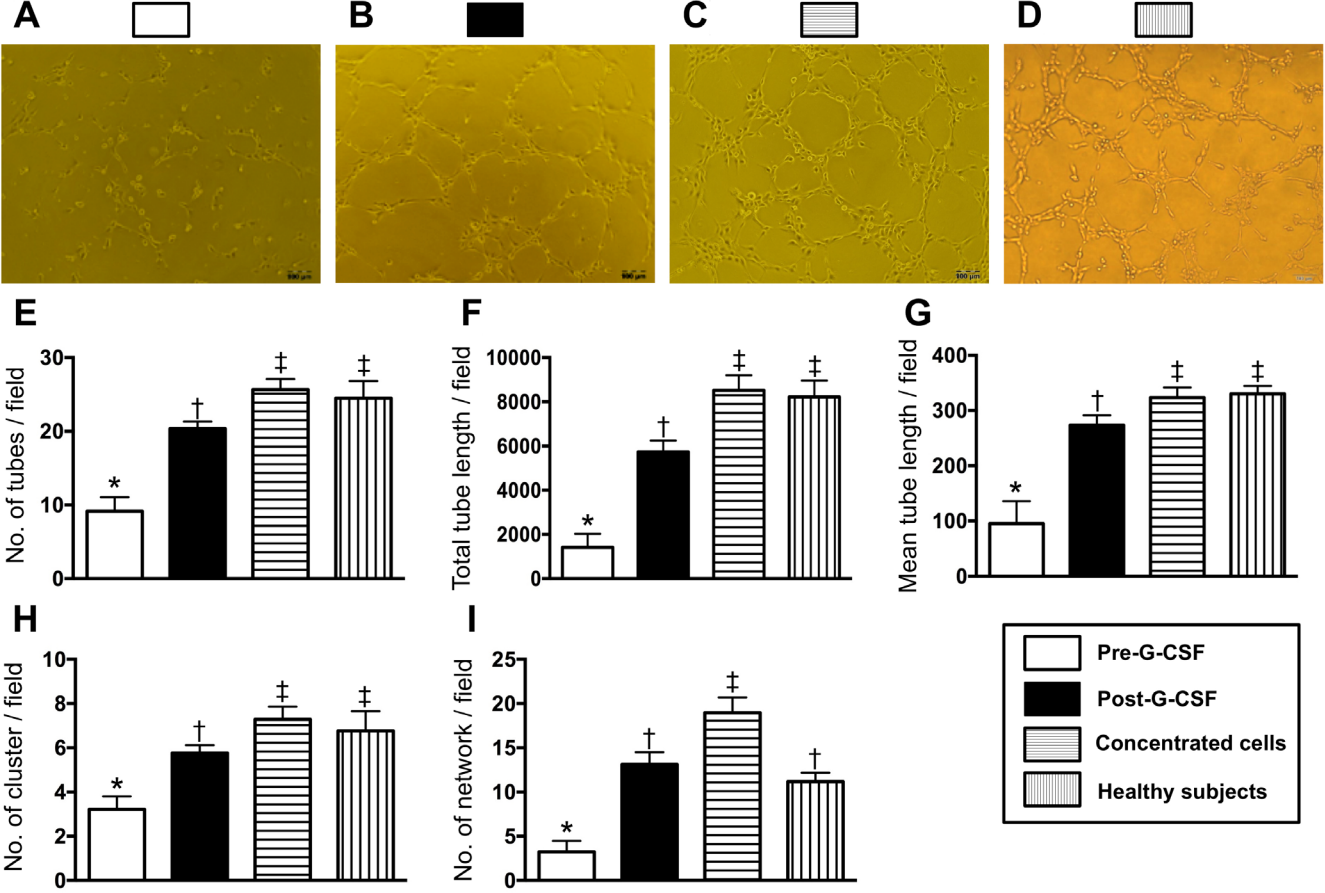
Angiogenesis capacity among the health-subjects and CDK-treatment patients prior to and after receiving granulocyte-colony stimulating factor (G-CSF) therapy (**A to D)** Illustrating the results of Matrigel assay of angiogenesis ability in CKD-treatment group prior to (A) and after G-CSF treatment (B) and from isolation of concentrated CD34+ cells (C), and in health-subjects (D), respectively. (**E)** Number of tubular formation, * vs. other groups with different symbols (†, ‡)*p* < 0.001. (**F)** Total tubular length, * vs. other groups with different symbols (†, ‡)*p* < 0.0001. (**G)** Mean tubular length, * vs. other groups with different symbols (†, ‡), *p* < 0.0001. (**H)** Number of cluster formation, * vs. other groups with different symbols (†, ‡)*p* < 0.001. **(I)** Number of network formation, * vs. other groups with different symbols (†, ‡)*p* < 0.001. Statistical analysis using Friedman test followed by Wilcoxon's signed-rank test with multiple comparison procedure. Symbols (*, †, ‡) indicate significance (at 0.05 level).

**Figure 6:**
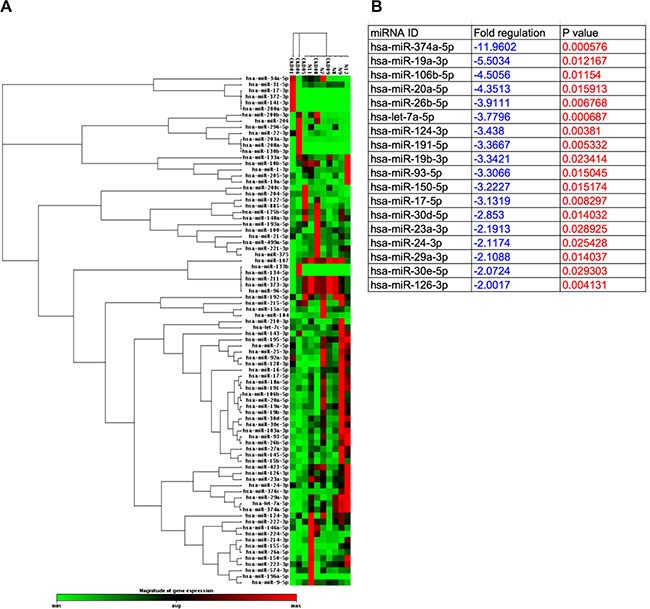
Clustergram illustrated that Total 84 miRNAs as candidates to be screened, were involved in one array by RT-qPCR

**Figure 7:**
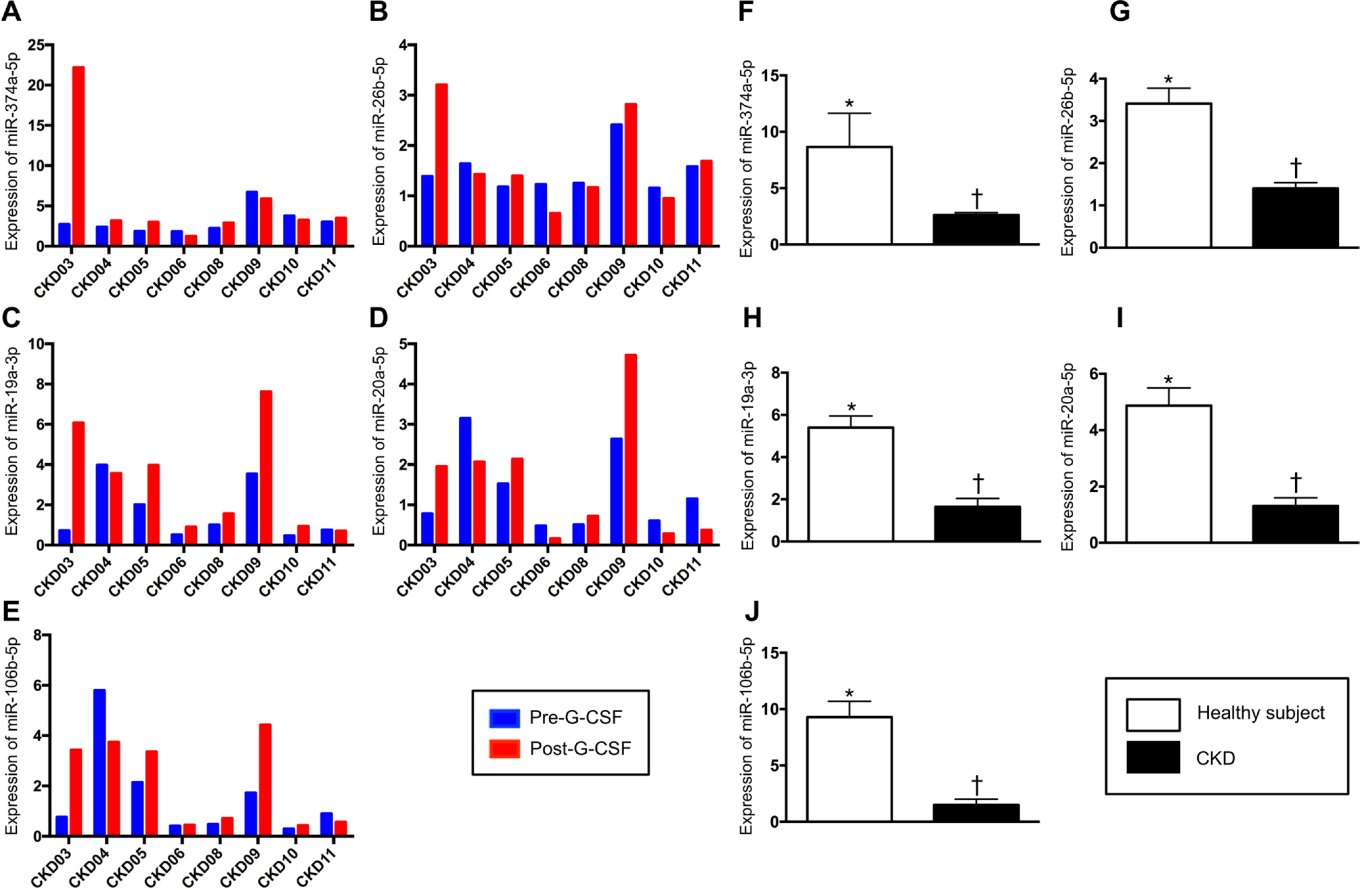
Comparison of circulating microRNA expressions between health-control subjects and CKD-treated patients (**A** to **E**) Illustrating the expressions of circulating levels of five miRNAs: miR-374a-5p, miR-19a-3p, miR-106b-5p, miR-26b-5p, miR-20a-5p in individual patient prior to and after receiving G-CSF treatment. (**F**) Analytical result of miR-374a-5p level, * vs. ^†^*p* < 0.001. (**G**) Analytical result of miR-26b-5p, level, * vs. ^†^*p* < 0.001. (**H)** Analytical result of miR-19a-3p level, * vs. ^†^*p* < 0.001. **(I)** Analytical result of miR-20a-5p level, * vs. ^†^*p* < 0.001. (**J)** Analytical result of miR-106b-5p level, * vs. ^†^*p* < 0.001.

## DISCUSSION

To the best of our knowledge, this was the first phase I clinical trial investigating the safety of autologous transfusion of CD34+ cells into the renal artery. The results of this clinical trial revealed several striking clinical implications. First, intra-renal transfusion of peripheral blood-derived autologous CD34+ cell was 100% safe and one-year survival rate was 100%. Second, creatinine level, an indicator of renal function, did not show notable change at the end of study period as compared with the baseline level. Third, the positive net change in creatinine level between the end of study period and baseline was relatively higher in CDK-control group than in CKD-treated group. Fourth, the anti-apoptotic miRNAs were significantly impaired in CDK-treated group as compared with that of healthy counterpart.

The most important finding in the present study was that the procedure of CD34+ therapy was 100% success with 0% complication rate, and all patients were uneventfully discharged and regularly followed up at outpatient department. Additionally, one-year survival rate was 100% in the study patients. Moreover, the renal ultrasound examination showed no tumorigenesis in right kidney of every patient. Accordingly, the findings of our study support that that autologous transfusion of CD34+ cells to CKD patients is safe.

It is a common medical issue that the renal function will progressively deteriorate in patients with CKD even with an optimal therapeutics [40], especially in patients with CKD stage ≥ III during one-year follow-up. An essential finding in the present study was that the creatinine level, an indicator of renal function, at the end of study period (i.e., at the 12^th^ month after CD34+ cell therapy) was identical to that at the baseline in CKD-treated group. On the other hand, in CKD-control group the creatinine level at the end of study period was notably increased than that at baseline. These findings suggested that CD34+ cell therapy might offer some effect on mitigating the deterioration of renal function in CKD patients.

A principal finding in the present study was that the circulating levels of micro-RNAs (i.e., miR-374a-5p, miR-19a-3p, miR-106b-5p, miR-26b-5p and miR-20a-5p, five indicators of proliferation, migration and anti-apoptosis of endothelial cells), were notably reduced in CDK-treated group as compared with health-control group. Additionally, Matrigel assay showed that prior to G-CSF treatment the angiogenesis ability was also markedly reduced in CKD-treated group as compared with the health-control group. Furthermore, the baseline circulating number of EPCs was remarkably lower in CKD patients than in health-control subjects. These findings highlighted that not only the number of EPCs in circulation but also integrity of the endothelial function and capacity of endothelial-cell repairmen/renew were substantially weakened in CKD patients. Interestingly, our recent study has shown that the circulating levels of EPCs and the angiogenesis ability in both *in-vitro* and angiographic studies were significantly reduced in patients with diffuse coronary artery disease (CAD) [39]. Therefore, the conclusions reached in our recent study [39] corroborated the findings in the present study. Importantly, the finding of our recent study [39] demonstrated that peripheral blood-derived CD34+ cell therapy significantly improved angina, heart failure and left ventricular function, supporting our hypothesis that CD34+ cell therapy might be beneficial to CKD patients with endothelial cell dysfunction.

One interesting finding in the present study was that flow cytometric analysis showed that EPC numbers (i.e., KDR+/CD34+/CD45-, CD133+/CD34+/CD45-, and CD34+) were not only significantly increased in renal vein than in circulation, but were also identified to be continuously drained from the vein into the circulation. Intriguingly, our recent study also exhibited that after intra-coronary transfusion of autologous CD34+ cells to the diffuse CAD patients, the numbers of EPCs were significantly higher in coronary sinus than in circulation [39]. These findings, in addition to being consistent with the conclusions reached in our recent report [39], suggest that the level of EPCs, maintained at a higher level inside renal arteries than that in circulation, might play an important role for endothelial cell repair and angiogenesis/neovascularization.

### Study limitation

This study has limitations. Frist, the number of sample size was small in this phase I clinical trial. However, this clinical trial was the first one in the world and the finding was attractive and promising. Accordingly, our findings endowed us with confidence to perform randomized and controlled double blinded phase II clinical trial of CD34+ cell therapy for CKD patients in our institute. Second, the IRB of Ministry of Health and Welfare strongly recommended that the CD34+ cell therapy was only permitted for only one renal artery. Therefore, we did not know whether the therapeutic effect of CD34+ cells on improving renal function would be better if two renal arteries were considered.

In conclusion, this was the first clinical trial by using transfusion of peripheral blood-derived autologous CD34+ cells into the right renal artery and the results showed that such a therapy was not only safe but also was able to maintain a stationary phase of the renal function during one-year follow-up.

## MATERIALS AND METHODS

### Ethics and study design

This clinical trial was approved by the Ministry of Health and Welfare, Taiwan, Republic of China (IRB No: 1040007105) and the Institutional Review Committee on Human Research at Chang Gung Memorial Hospital (IRB No: 102–0358A) in 2014 and conducted at Kaohsiung Chang Gung Memorial Hospital, a tertiary referral center. This study was funded by research grants from both the National Science Council, Taiwan, Republic of China (104-2325-B-182A-004 NMRPG8E0021) and Chang Gung Memorial Hospital, Chang Gung University (Grant number: CMRPG8D1231 and CMRPG8D1232).

This was a phase I clinical trial with prospective enrollment of 10 consecutive CKD patients to essentially (i.e., the first priority) test the safety of right intra-renal artery transfusion of circulation-derived autologous CD34+ cells (5.0 × 10^7^) at a single medical center.

### Inclusion and exclusion criteria

Inclusion criteria included patients (> 20 yrs old and < 80 yrs old) having CKD stages III and IV with documented history of hypertension prior to identification of CKD and receiving regular and optimal anti-hypertension medications, including angiotensin converting enzyme inhibitors (ACEI), angiotensin II type I receptor blocker (ARB) agents, calcium channel blocker agents, or beta-blocker agents. Additionally, the serum creatinine level and creatinine clearance rate (Ccr) maintained within 30% of the baseline in recent 6 months. Furthermore, patients were willing to participate in the clinical trial.

Patients with history of the following conditions were excluded from the study: Hepatitis B or C carrier, surgery, trauma, or myocardial infarction within the preceding 3 months, liver cirrhosis, hematology disorders, CKD stage V (defined as creatinine clearance < 15 mL/min) or stage I and stage II, malignancy, febrile disorders, acute or chronic inflammatory disease at study entry, severe mitral or aortic regurgitation, congestive heart failure (NYHA Fc ≥ 3), expected life expectancy < 2.0 yrs, age < 20 yrs or ≥ 80 yrs, or pregnant women.

Between November 2014 and August 2015, patients meeting the above criteria were assessed for eligibility at the institute. Over a period of 10 months, 27 consecutive patients with CKD were screened. Sixteen (59.3%) of the 27 patients were excluded due to hesitation or refusal (i.e., screen failed). The remaining 11 patients were enrolled. One (9.0%) of these 11 patients regretted later to participate in the study after screening and subsequently withdrew from the clinical trial (i.e., early termination without receiving CD34+ cell treatment). Accordingly, 10 patients were finally enrolled into the study within 10 months. For monitoring the safety, one patient per month receiving CD34+ cell treatment was clinically followed, as strongly requested by the Ministry of Health and Welfare, Taiwan, Republic of China.

### Procedure and protocol for isolation of autologous CD34+ cells and intra-renal artery transfusion

The procedure and protocol for isolation of circulatory CD34+ cells were based on our recent reports [39]. In detail, prior to the isolation of the peripheral blood derived CD34+ cells, granulocyte-colony stimulating factor (G-CSF) (5 μg/kg, q12h for 8 doses) was subcutaneously given to each patient to augment the number of circulatory CD34+ cells for facilitating subsequent collection via leukapheresis. After the last dose of G-CSF, the mononuclear cell preparation isolated during leukapheresis was enriched for CD34+ cells by using a commercially available device [COBE Spetra 6.1 (Terumo BCT, INC.)] at 8:00 a.m. through a double lumen catheter inserted into the right femoral vein. At a procedure time about four hours, adequate number of blood derived CD34+ cells were isolated (purity through fluorescence-activated cell sorting for CD34+ cells) and ready for intra-renal artery transfusion.

Based on the International Society of Hematotherapy and Grafting Engineering (ISHAGE) Guidelines for CD34+ cell determination by flow cytometry to quantitate CD34+ cell in circulation, hematological stem cells were characterized by the presence of the surface markers CD34^high^/CD45^dim^/SSC^low^ that were utilized to quantify the number of isolated CD34+ cells. The formula for calculating the number of peripheral blood derived CD34+ cells: Number of CD34+ cells = (Percentage of CD34+ cells) x WBC count x 10^3^ x peripheral-blood stem cell (PBSC) volume (mL). In the current study, the flow cytometric analysis was performed according to the College of American Pathology current guideline with a performance coefficient of variation (CV): < 4.0% (3.4 ± 2.5) (by definition of < 10.0% is acceptable).

After completing the CD34+ cell collection, the patients were immediately sent to cardiac catheterization room for receiving the right intra-renal CD34+ cell transfusion. Transradial arterial approach and carefully engagement of right renal artery was performed for each patient for renal arterial angiographic study, followed by CD34+ cell slow transfusion via a guiding catheter. In the present study, to avoid contrast media-induced nephropathy, only about 5.0 to 6.0 cc of contrast media was utilized for each renal angiographic study for identifying the anatomy of the right renal artery. Additionally, the puncture of right internal femoral vein and engagement of right renal vein by guiding catheter were performed for measuring the serial changes of EPCs in renal vein.

### Laboratory assessment of levels of EPC in circulation and renal vein by flow cytometry

EPCs in peripheral and renal vein blood were identified by flow cytometry using double staining as depicted in our recent report [39] using a fluorescence-activated cell sorter (FACSCalibur™ system; Beckman Coulter Inc, Brea, CA). Every analysis included 300,000 cells per sample. The assays for circulatory and coronary sinus EPCs in each sample were performed in duplicate and mean levels were reported. Intra-assay variability based on repeated measurement of the same blood sample was low with a mean CV of 3.9% study subjects.

One blood sample was drawn at 8:00 a.m. prior to G-CSF injection and the other was collected following G-CSF treatment for flow cytometric analysis. In addition, to elucidate the serial changes in the levels of EPC in renal vein, a series of blood samples were drawn from the renal vein at 0 minute prior to CD34+ cell transfusion and at 5, 10, and 30 minutes after CD34+ cell transfusion for flow cytometric analysis.

### Collection of peripheral blood, culture of endothelial progenitor cells, and angiogenesis measurement by matrigel assay

For determining the angiogenesis ability, peripheral blood (10 cc) was drawn in each CDK patient and healthy subject prior to G-CSF administration. The isolated mononuclear cells from peripheral blood were cultured in a 100 mm diameter dish with 10 mL DMEM culture medium containing 10% FBS. By 21-day culturing, abundant cobblestone-like cells, a typical feature of EPCs, were obtained from each study subject. Flow cytometric analysis was performed for identification of cellular characteristics (i.e., EPC surface markers) with appropriate antibodies on day 21 of cell cultivation.

To evaluate whether there was different angiogenesis capacity between healthy group and CKD group, peripheral blood-derived EPCs (1.0 × 10^4^ cells) (*n* = 10 per group) were incubated on 96-well plates at 1.0 × 10^4^ cells/well in 150 μL serum-free M199 culture medium mixed with 50 μL cold Matrigel (Chemicon international) for 3-hour incubation at 37°C in 5% CO_2_. Three random microscopic images (200x) were taken at each well for counting cluster, tube, and network formations with the mean values derived (Figure 5).

### Assessment of blood urea nitrogen (BUN), creatinine and urine protein levels

Blood samples were serially collected before and after the autologous transfusion of CD34+ cells into the renal artery [i.e., prior to, and at days 2 and 3 (i.e., at days 6 and 7 of hospitalization) after, as well as at 1 week and 1st, 3rd, 6th, 9th and 12th months after the CD34+ cell transfusion) for assessment of serum levels of creatinine (mg/dL) and BUN (mg/dL). Additionally, the ratios of urine total protein (uTP) and urine albumin (uAL) to urine (uCre) creatinine ratio were also serially measured with the same intervals in measuring creatinine and BUN.

Concentrations of serum creatinine and BUN, and urine protein level (one spot urine was utilized) were measured by the standard method in the Department of Clinical Biochemistry and Pathology of our hospital.

### MicroRNAs extraction and quantitative analysis for determining the integrity of endothelial function

Total rna extracted from plasma and cells using the miRNeasy kit (Qiagen) was based on the protocol of the manufacturer. C. elegans miR-39 (cel-miR-39-3p) mimics were loaded in plasma as Spike-in control. For mature miRNA quantification, cDNA was generated with reverse transcription using miScript II RT kit (Qiagen) and Cdna, which was further utilized as a template for real-time PCR. Expressions of human miR-374a-5p, miR-19a-3p, miR-106b-5p, miR-26b-5p and miR-20a-5p were then quantified by miScript SYBR Green PCR assay (Qiagen), and finally were normalize by cel-miR-39-3p in plasma and RNU6 in cells (Qiagen), respectively. Triplicate assays were performed for each sample on Step One-Plus machine (ABI).

### Medication during the CD34+ cell transfusion

Heparin (3000 IU) was intra-arterial given to each patient at the beginning of the procedure and its effect was immediately reversed by intra-venous 20 mg of protamine after the CD34+ cell transfusion.

### Clinical and 12-month follow-up

In addition to regular follow-up of each patient at our outpatient clinic, a case report form that recorded all clinical information of the patient, including the presence or absence of acute or sub-acute events, was designed for each patient and completed by a research nurse regularly after each visit and on readmission as well as irregular telephone interviews.

### Statistical analysis

All values are expressed as the mean ± SD, number, or percentage, as appropriate. Differences in continuous variables between two groups were analyzed by *t* test or chi-square test, when it is appropriate. Continuous variables at different time points in each group were compared using Friedman test followed by Wilcoxon's signed-rank test with multiple comparison procedure. Statistical analysis was performed using SPSS statistical software for Windows Version 24 (SPSS for Windows, Version 24; SPSS, IL, U.S.A.). A value of *p* < 0.05 was considered statistically significant.
